# Experience has a limited effect on humans’ ability to predict the outcome of social interactions in children, dogs and macaques

**DOI:** 10.1038/s41598-020-78275-5

**Published:** 2020-12-04

**Authors:** Sasha Donnier, Gyula Kovács, Linda S. Oña, Juliane Bräuer, Federica Amici

**Affiliations:** 1 Fundació UdG: Innovació I Formació, Universitat de Girona, Carrer Pic de Peguera 11, 17003 Girona, Spain; 2 Institute of Psychology, Friedrich Schiller University Jena, Leutragraben 1, 07743 Jena, Germany; 3 Max Planck Research Group ‘Naturalistic Social Cognition’, Max Planck Institute for Human Development, Berlin, Germany; 4 Max-Planck-Institute for the Science of Human History, Jena, Germany; 5 Department of Human Behavior, Ecology and Culture, Research Group “Primate Behavioural Ecology”, Max Planck Institute for Evolutionary Anthropology, Leipzig, Germany; 6 Institute of Biology, Behavioral Ecology Research Group, University of Leipzig Faculty of Life Science, Leipzig, Germany

**Keywords:** Coevolution, Animal behaviour

## Abstract

The ability to predict others’ behaviour represents a crucial mechanism which allows individuals to react faster and more appropriately. To date, several studies have investigated humans’ ability to predict conspecifics’ behaviour, but little is known on our ability to predict behaviour in other species. Here, we aimed to test humans’ ability to predict social behaviour in dogs, macaques and humans, and assess the role played by experience and evolution on the emergence of this ability. For this purpose, we presented participants with short videoclips of real-life social interactions in dog, child and macaque dyads, and then asked them to predict the outcome of the observed interactions (i.e. aggressive, neutral or playful). Participants were selected according to their previous species-specific experience with dogs, children and non-human primates. Our results showed a limited effect of experience on the ability to predict the outcome of social interactions, which was mainly restricted to macaques. Moreover, we found no support to the co-domestication hypothesis, in that participants were not especially skilled at predicting dog behaviour. Finally, aggressive outcomes in dogs were predicted significantly worse than playful or neutral ones. Based on our findings, we suggest possible lines for future research, like the inclusion of other primate species and the assessment of cultural factors on the ability to predict behaviour across species.

## Introduction

Predicting is a non-conscious and non-stop cognitive process, in which expectations about the future are formed and compared to the actual events of the sensory environment^1–3^. The ability to predict others’ behaviour is an evolutionary mechanism which plays a crucial role in our lives. Humans, indeed, can not only predict the consequences of their own actions (‘egocentric framework’), but also others’ behaviour (‘allocentric framework’)^4^. By predicting how others will behave, we can more quickly identify and react to dangers^5,6^, reduce our reaction times^2,3,7,8^, and anticipate the consequences of others’ actions more accurately^9,10^, which might have resulted in a selective advantage in our evolutionary history. To date, several studies have investigated humans’ ability to predict conspecifics’ behaviour, but little is known on our ability to predict behaviour in other species.

One indirect way to approach this issue has been the study of how humans recognize emotional expressions in other animals. Across species^11–15^, emotions are often expressed through behavioural and somatic displays which serve as signals for other individuals and may have a crucial communicatory and social function^16–19^. By reading emotional expressions, humans can use cues that allow them to predict how others will behave, thus gaining important fitness benefits^18,19^. As emotions may be displayed differently across taxa, however, cross-species emotion recognition may be especially challenging^20^, although different factors may facilitate the emergence of this ability in evolutionary or developmental terms. In evolutionary terms, for instance, some species might share a long history of close association, so that the fitness benefits of reading each other’s emotions and predicting each other’s behaviour may be especially high and thus selected through evolution (i.e. co-evolution)^21–26^. Reciprocal adaptation is therefore crucial to identify co-evolution, as both species constitute an important selective pressure for each other, and consequently evolve specific behavioural and cognitive traits^24,27,28^. In developmental terms, instead, individuals may acquire the relevant skills to read the emotions of other species and predict their behaviour through direct experience with them^29–32^. Moreover, recognizing emotions in some species might simply be easier if these share physical and functional traits allowing to produce homologous expressions, perhaps as the result of phylogenetic closeness^33,34^.

Humans’ ability to recognize emotions in other species has indeed been intensively studied in domestic dogs (*Canis familiaris*), which share with us a long history of close association since their domestication, at least 30,000 years ago^35,36^. These studies lead to a somewhat complicated picture. Humans, for instance, can recognize dog emotions based on their auditory signals effortlessly^30,37–40^. However, children and adults do not always understand the body signals or actions of dogs correctly^41,44^. Moreover, humans more easily recognize positive dog emotions (e.g. happiness) when using visual stimuli^32,41,45,46^, while often confusing their negative emotions with each other (e.g. fear^32,41,45,47^; but see^46^). Therefore, support for the co-evolution hypothesis in dogs and humans is to date scant.

The role played by experience on cross-species emotion recognition is even more controversial. In some studies, inexperienced humans (i.e. those having no dogs in their household; non-owners) could successfully read positive and negative dog emotions^29,31^, even better than humans who had extensive personal experience with dogs (e.g. dog owners^41^). In other studies, however, the ability to recognize dog emotions did not differ between dog-owners and non-owners^38,39,46^, despite partially increasing with age and experience in some cases and for certain emotions^29–32^; but see^48^.

Overall, these studies provided important information on the human ability to read dog emotions, but do not allow to draw reliable conclusions on our ability to predict dog behavior per se. Studies of emotion recognition, for instance, often present still pictures as visual cues^31,41,49^ (but see^32,45,46^). During everyday interactions, however, humans can rely simultaneously on more cues to predict others’ behaviour, so that performance during emotion recognition tasks may underestimate actual human predicting abilities. Moreover, experience may play a stronger role when predicting others’ behaviour, as it increases the repertoire of familiar, recognizable and predictable actions by others^50,51^, and the ability to infer their abilities and inner states^52^. Furthermore, understanding how humans predict the behaviour of other species (rather than simply reading their online emotions) has crucial practical implications as well. Experience with animals may decrease the risk of being injured^48,53–58^, for instance, by increasing the ability to predict their behaviour, something especially important to prevent injuries from dogs and other species^56^.

In the current study, we aimed to specifically test humans’ ability to predict behaviour in humans as well as in two non-human species, and the role of experience and evolution. For this purpose, we presented participants with short videoclips of real-life social interactions in dog, child and non-human primate (NHP) dyads, and then asked them to predict the outcome of the interactions observed (i.e. aggressive, neutral or playful). Participants were selected according to their prior specific experiences, having had extensive experience either with dogs, children, NHPs or with none of these species. We included NHPs to better disentangle the potential effects of experience and evolution on the ability to predict social interactions. In particular, the inclusion of NHPs allowed us understanding whether humans’ ability to predict dog behaviour is species-specific (e.g. as a result of co-evolution between humans and dogs), or rather common to other non-human species (and perhaps more generally dependent on the species-specific experience with the species). Therefore, if inter-specific co-evolution is linked to an enhanced ability to predict behaviour in other species, every participant should predict dog behaviour better than NHP behaviour (as humans co-evolved with dogs, but not NHPs), and similarly to child behaviour (Prediction 1). If, however, experience with one species is linked to an enhanced ability to predict its behaviour, participants with species-specific experience should predict the behaviour of the species on which they have experience better, as compared to participants with no specific experiences (Prediction 2). Finally, we expected that participants would predict aggressive outcomes better than playful and neutral ones (Prediction 3), as the ability to predict potentially dangerous outcomes may have a greater adaptive function, especially in other species; see^5,8,47,49^.

## Methods

### Ethical statement

The study was approved by the ethical committee of the University of Jena and of the Max Planck Society and was carried out in accordance with the relevant guidelines and regulations. Participants took part on a completely voluntarily base and were not rewarded for taking part in the experiments. Informed consent was obtained from all subjects and also for subjects under 18, from their parent and/or legal guardian.

### Participants

We tested 77 white participants (53 females, 24 males; mean age ± SD = 28.3 ± 11.3 years; 52 from Germany, 3 from Italy, 11 from France, 9 from Spain, 1 from Great Britain and 1 from Belgium). Participants were selected according to their experiences with one of the three species (dogs, human children, NHPs), which had to be both extensive (more than 1-year continuous experience with daily or almost daily contact, for either personal or working reasons) and exclusive (i.e. extensive experience had to be limited to no more than one of the three tested species). We recruited 27 participants with dog experience (22 females, 5 males; mean age ± SD = 25.9 ± 10.8), 17 with child experience (11 females, 6 males; mean age ± SD = 34.3 ± 13.7), 14 with NHP experience (9 females, 5 males; mean age ± SD = 27.4 ± 3.8), and 19 with no experience with any of these species (12 females, 7 males; mean age ± SD = 26.1 ± 11.8). In particular, participants with NHP experience had extensive and exclusive work-related experience with group-living primates (i.e. socially housed or wild primates). For this group of participants no strict species-specific experience was required, although most participants had experience with macaques. Participants were recruited in Germany, France, Spain and Great Britain through word of mouth, social networks and active recruitment in the facilities and libraries at the University of Jena (Friedrich-Schiller-Universität), Germany. Before starting a testing session, participants gave their informed consent.

### Materials and procedure

We prepared 54 video clips (18 per species). Each video displayed two conspecifics (i.e. either two dogs, two NHP or two children) that could either interact in an aggressive way, in a playful way, or neutrally. The video consisted of two parts, and only the first one was shown to the participants. The first part of the video, which was 2- to 5-s long, was interrupted 10 frames before the interaction took place, and could thus end in three different ways: (i) one of the two individuals made an aggressive signal/action toward the other (e.g. stiff body posture in dogs, open mouth in NHP, suddenly moving toward the partner in children); (ii) one of the two individuals made a playful signal/action toward the other (e.g. play bow in dogs, play face in NHP, smile in children); (iii) no obvious/apparent signal/action was displayed. The second part of the video, which was not shown to participants and lasted up to 17 s, depicted the aggressive, playful or neutral interaction and its outcome (e.g. one child pushed the other away, children played together, children did not interact with each other). All videos were presented together with sound (i.e. they had non-verbal vocalizations and voices) but no videos of the children included intelligible speech (Supplementary Video 1).

The video clips were selected by three different researchers with previous extensive experience working with dogs, humans and NHPs (JB, LSO, FA). Moreover, 3 further experts for each species, blind to the aims of the study, coded the outcome of each video independently, confirming the original selection (i.e. 83% agreement across species and videos). Videos of dogs included different breeds video-recorded while freely interacting in gardens or dog parks. Videos of children included pre-linguistic children as well as pre-teens (1–10 years-old). Videos of macaques included free-ranging Barbary macaques (*Macaca sylvanus*) housed at Kintzheim, in France, as video-recorded during natural interactions in their group.

To reduce testing time and maintain participants’ motivation, the 54 videos were split in two groups, and each participant only watched videos from one of these groups (9 videos per species, 3 for each possible outcome). The order of videos was randomized across participants. Participants were either tested on a computer screen in our laboratory or via online presentation (Skype) at the home computer of the participants. Participants were then given a Word document with questions on their previous experiences with dogs, NHPs or children, and other biographical information (e.g. age, gender). As the file had been filled in, they were given a PowerPoint presentation consisting of one video per slide (which they could watch a maximum of two times), followed by a slide with proposed answers (i.e. whether the outcome of the video watched was aggressive, playful or neutral). Participants had to note the selected response into a Word document. At the end of the testing session, participants also rated their confidence about their performance on a 7-grade Likert-type scale, separately for each species. The experimenter ensured that participants followed the procedures (e.g. watching each video no more than twice) by staying close to participants being tested live, or by sharing the screen with the participants tested via Skype.

### Statistical analyses

To analyze data, we used generalized linear mixed models (GLMMs^59^) with the glmmTMB package (version 1.0.1^60^) in R statistical software (R Core Team, version 3.5.0). In particular, we tested whether the probability to predict the outcome of each video correctly (as binomial response) was predicted by the 3-way within subject design with species (3 levels: dog, human, NHP), outcome (3 levels: aggressive, playful, neutral) and previous experience (4 levels: dogs, children, NHP, none) as factors. We further included participants’ age, sex and trial number as control factors, and participant and video identity as random factors.

We *z*-transformed continuous variables (i.e. participants’ age and trial number) to facilitate model convergence and interpretation of model coefficients. We used likelihood ratio tests^61^ to compare full models containing test, control predictors and random factors, with null models containing only control predictors and random factors using the ANOVA function of R. When full models differed significantly from null models, likelihood ratio tests were conducted to obtain the *p* values for each test predictor via single-term deletion using the R function “drop1”^62^. If the 3-way interaction was not significant, we re-ran the model only including the significant 2-way interactions and the main terms. In case of significant categorical test predictors with more than two categories, we conducted post-hoc comparisons using Tukey tests, which we only report if significant. To rule out collinearity, we determined the Variance Inflation Factors (VIFs; see^63^), which were found to be minimal (maximum VIFs across all models = 1.0). We also checked the distribution of the residuals using the functions testDispersion and simulateResiduals of the R package DHARMa^34^, which did not indicate any deviation from the expected distribution. We detected no convergence issues in the models. Finally, we run Spearman exact correlations, separately for each species, to assess if confidence ratings correlated (i) with previous species-specific experience, and (ii) with performance in the task.

## Results

Table 1 provides information on the percentage of trials that were correctly answered by participants with different species-specific experience. Table 2 summarizes the responses provided by participants when they failed to predict the outcome of the behaviours observed.

**Table 1:** For each species and outcome observed in the video, percentage of trials (± SD) in which participants with extensive species-specific experience provided a correct response.

Species and outcome observed	Experience	Percentage of correct responses
**Children**		
Aggressive	Yes	69 ± 47
	No	68 ± 47
Playful	Yes	56 ± 50
	No	66 ± 48
Neutral	Yes	72 ± 46
	No	65 ± 48
**Dogs**		
Aggressive	Yes	43 ± 50
	No	41 ± 49
Playful	Yes	69 ± 46
	No	79 ± 41
Neutral	Yes	51 ± 50
	No	45 ± 50
**NHPs**		
Aggressive	Yes	86 ± 35
	No	68 ± 47
Playful	Yes	76 ± 43
	No	54 ± 50
Neutral	Yes	83 ± 38
	No	79 ± 41

**Table 2:** For each species and outcome observed in the video, percentage of wrong trials in which participants provided one of the possible wrong responses.

Species and outcome observed	Wrong response given	Percentage of wrong responses
**Children**		
Aggressive	Playful	12
	Neutral	88
Playful	Aggressive	48
	Neutral	52
Neutral	Aggressive	50
	Playful	50
**Dogs**		
Aggressive	Playful	49
	Neutral	51
Playful	Aggressive	54
	Neutral	46
Neutral	Aggressive	26
	Playful	74
**NHPs**		
Aggressive	Playful	32
	Neutral	68
Playful	Aggressive	63
	Neutral	38
Neutral	Aggressive	60
	Playful	40

**Figure 1:**
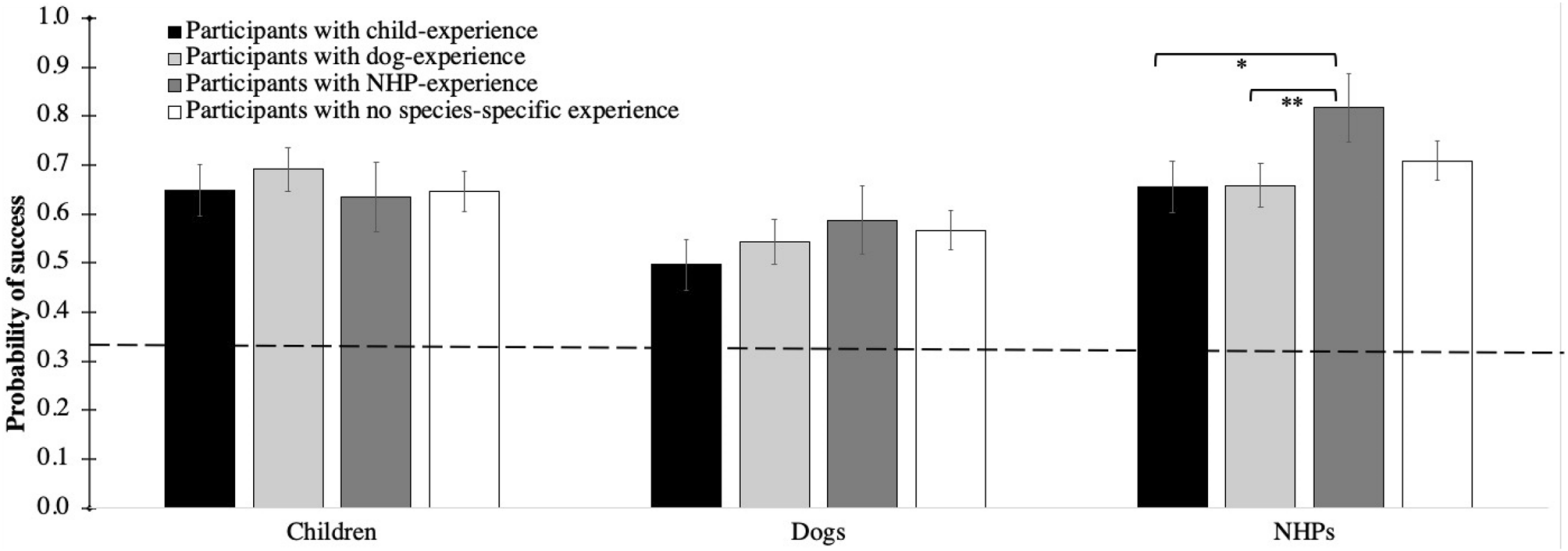
For each species and previous experience, mean (± SE) probability of successfully predicting the outcome of the videos (after collapsing all three possible outcomes: aggressive, playful, neutral). Significant differences were assessed with post-hoc tests and are marked with asterisks in the figure (*p < 0.050; **p < 0.005). Horizontal dashed line marks chance level performance, and NHPs stands for non-human primates.

**Figure 2:**
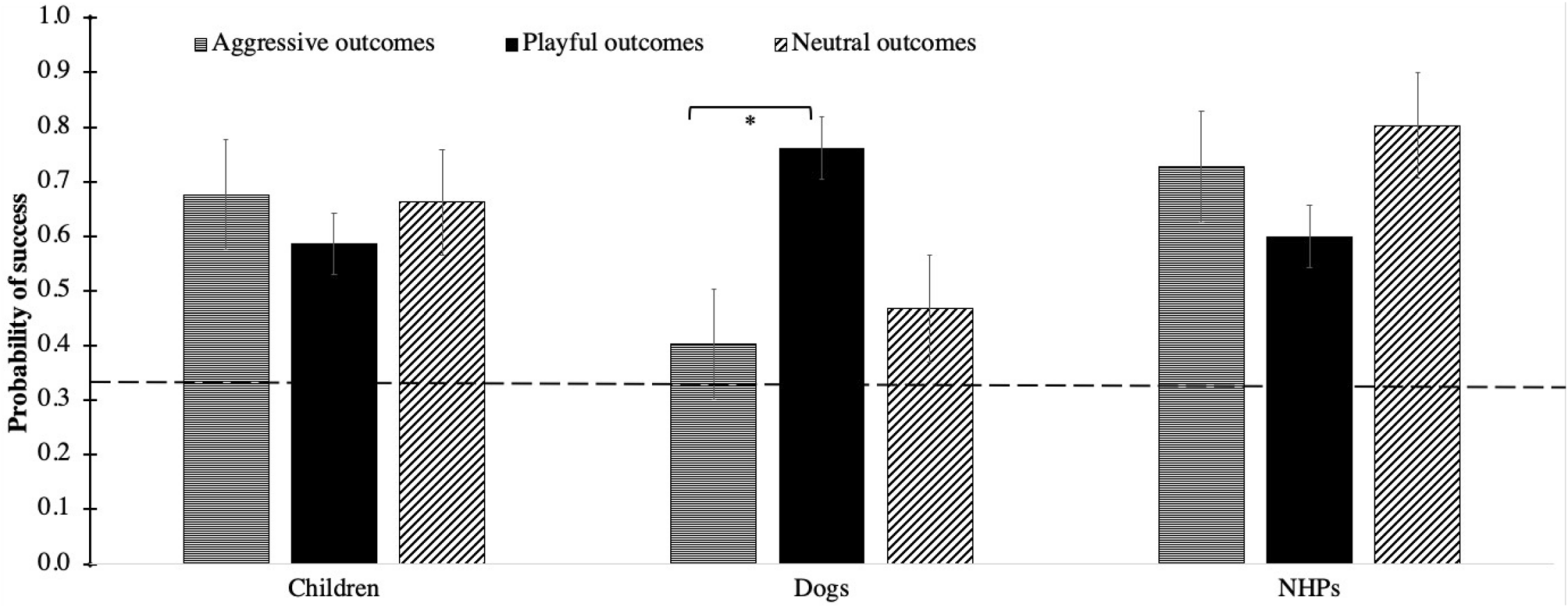
For each species and possible outcome, mean (± SE) probability of successfully predicting the outcome of the videos. Significant differences were assessed with post-hoc tests and are marked with an asterisk in the figure (*p < 0.050). Horizontal dashed line marks chance level performance, and NHPs stands for non-human primates.

The full model significantly differed from the null model (LRT: *χ*^2^ = 51.02, df = 35, *p* = 0.039). The 2-way interactions of experience with species (*p* = 0.038) and outcome with species (*p* = 0.014) were significant (Table 3; Figs. 1, 2). In particular, participants with NHP experience had a higher probability of correctly predicting the outcome of NHP videos as compared to participants with dog experience (*p* = 0.003) and child experience (*p* = 0.019; Fig. 1). In dogs, moreover, participants had a higher probability to predict playful outcomes than aggressive ones (*p* = 0.031; Fig. 2). No other significant differences were detected with post-hoc comparisons.

**Table 3:** Results of Model 1, including estimates, standard errors (SE), confidence intervals (CIs), likelihood ratio tests (LRT), degrees of freedom (df) and *p* values for each test and control predictor (in parentheses, the reference category).

Model 1	Estimate	SE	2.5% CI	97.5% CI	LRT	df	*P*
Intercept	1.53	0.53	0.5	2.57	–	–	–
Species (dog)	− 1.85	0.70	− 3.22	− 0.47	–	–	–
Species (NHP)	− 0.47	0.69	− 1.81	0.88	–	–	–
Outcome (neutral)	− 0.57	0.64	− 1.83	0.69	–	–	–
Outcome (playful)	− 0.49	0.65	− 1.77	0.78	–	–	–
Experience (dog)	0.17	0.26	− 0.33	0.67	–	–	–
Experience (NHP)	− 0.15	0.29	− 0.72	0.42	–	–	–
Experience (none)	− 0.03	0.28	− 0.58	0.51	–	–	–
*Participants’ age*	0.006	0.06	− 0.11	0.12	0.01	1	0.914
*Participants’ sex*	− 0.2	0.12	− 0.43	0.04	2.66	1	0.103
*Trial number*	0.04	0.05	− 0.07	0.14	0.51	1	0.474
Species (dog) *Outcome (neutral)	0.76	0.91	− 1.03	2.54	12.52	4	0.014*
Species (NHP) *Outcome (neutral)	1.51	0.91	− 0.26	3.28			
Species (dog) *Outcome (playful)	2.14	0.92	0.33	3.95			
Species (NHP) *Outcome (playful)	− 0.004	0.9	− 1.75	1.74			
Species (dog) *Experience (dog)	− 0.003	0.35	− 0.68	0.67	13.36	6	0.038*
Species (NHP) *Experience (dog)	− 0.25	0.35	− 0.94	0.44			
Species (dog) *Experience (NHP)	0.57	0.4	− 0.22	1.35			
Species (NHP) *Experience (NHP)	1.09	0.43	0.26	1.93			
Species (dog) *Experience (none)	0.35	0.37	− 0.38	1.08			
Species (NHP) *Experience (none)	0.32	0.38	− 0.43	1.07			

An asterisk denotes significant test predictors. Controls are in italics. Participants’ age and trial number had been previously *z*-transformed. NHP stands for non-human primate. Subject and video identity were included as random factors in the model.

Finally, individuals with prior experience with a given species showed higher confidence ratings for that species when compared to participants without experience. This was true for dogs (*ρ*_*s*_ = 0.41, n = 75, *p* < 0.001), NHP (*ρ*_*s*_ = 0.57, n = 75, *p* < 0.001) and children (*ρ*_*s*_ = 0.25, n = 75, *p* = 0.030). However, higher confidence ratings did not correlate with higher performance, in any of the species (dogs: *ρ*_*s*_ = 0.07, n = 75, *p* = 0.56; NHP: *ρ*_*s*_ = 0.17, n = 75, *p* = 0.14; children: *ρ*_*s*_ = 0.14, n = 75, *p* = 0.241).

## Discussion

In this study, we tested humans’ ability to predict the outcome of social interactions in dyads of dogs, humans and non-human primates (NHPs). Specifically, we aimed to disentangle the effects of evolution and previous experience with a given species on the ability to predict its behavioural outcomes. Our results provide no support to the co-evolution hypothesis, as participants did not predict the outcome of dog interactions better than NHP interactions. Moreover, species-specific experience had only a very limited effect on humans’ ability to predict the outcome of behavioural interactions, in that participants who had NHP experience were better at predicting the behavioural outcome of NHP interactions, but participants with dog experience or child experience were not better at predicting the behavioural outcome of dog or child interactions. Finally, we expected that participants would predict aggressive outcomes better than playful or neutral ones, as preventing potentially dangerous outcomes should have a greater adaptive function. However, the outcomes of all types of interactions were predicted similarly well for children and NHP. For dogs, however, participant’s ability to predict aggressive outcomes was significantly worse compared to playful outcomes. In the following, we will discuss each result and its implications in more detail.

As stated above, our study provides no support to the dog–human co-evolution hypothesis^21–26^ (but see below for further discussion). In contrast to our predictions, overall performance was lower when predicting the outcome of dog interactions than human and NHP interactions (see species estimates in Table 3). Therefore, our study provides no support to the hypothesis that, through evolution, humans would have evolved special skills to generally predict the social behavior of dogs. This is in line with abundant literature showing that children and adults often fail to correctly understand dog signals or behaviour^32,41–45,47^. Likely, the selective pressure exerted by humans on dogs has been much higher than the one exerted by dogs on humans: therefore, while dogs largely adapted to humans (with important changes in their behaviour and cognition^22,23,64^, there is no evidence that also humans evolved special skills as a result of co-evolution.

Furthermore, participants were surprisingly good at predicting the outcome of NHP interactions, even when having no prior experience with the species. Clearly, NHP are phylogenetically closer to humans than dogs, and thus share more physical and functional similarities^33,34^. This might allow NHP to produce homologous facial expressions^65–67^, which might have allowed participants to more easily predict the outcome of their interactions. However, the use of facial expressions seems to be specific to the communicative repertoire of the species^66,68^, with some being morphologically similar in humans and NHPs, but corresponding to different emotional states^68,69^; see^56^. Moreover, previous studies explicitly testing human ability to read NHP emotions with pictures revealed a relatively low performance (e.g.^49,56^), which contrasts with the results of the current study. Although direct comparisons between studies are difficult due to differences in the methodological approaches (e.g. in the number of different stimuli shown, and especially in the NHP included), participants’ success in our study might depend on the fact that more sources of information other than facial cues were available (e.g. NHP posture, context), which might have facilitated participants’ performance^70,71^.

Partially supporting our predictions, having previous experience with a given species had an effect on humans’ ability to predict the outcome of the interactions, but only for NHPs. Previous studies on dogs have found contrasting evidence on the effect of experience on humans’ ability to read dog emotions or predict their behaviour, with some studies showing a negative effect of experience in some contexts (e.g.^29,41^), some a positive one^29–32^, and some no differences^38,39,46^. Recently, Amici and colleagues (2019) proposed that the effect of experience might be more complex: rather than being affected by direct personal experience with dogs, the ability to read their emotions (and predict their behaviour) might increase when humans grow up in a cultural milieu with a positive attitude toward dogs^49^. Therefore, to control for cultural effect in future studies, it will be important to compare participants across different cultures in their ability to predict the outcome of dog interactions.

Furthermore, experience had a significant effect on participants’ ability to predict the outcome of NHP interactions. This effect of experience confirms previous findings in macaques with static pictures as stimuli^56^. At the moment, it is unclear why experience should increase participant’s performance for NHP, but not for dogs. In this study, participants with dog experience included dog owners and individuals with work experience on dogs. In contrast, participants with NHP experience only included individuals with work experiences. Possibly, working with a species provides a more specific and extensive expertise, as compared to sharing the daily life with an animal (see e.g.^41^). Therefore, future studies have to disentangle how different kinds of experience might affect humans’ ability to predict social behaviour in other species – something that might be especially important to decrease the frequency of potentially dangerous interactions with dogs^72,73^ and NHPs^74^ in every-day life.

The lack of a significant effect of experience on the ability to predict the outcome of child interactions is not entirely unexpected. On the one hand, it is possible that our ability to predict conspecifics’ behaviour is largely innate^75,76^. On the other hand, it is likely that all of our participants had cumulated extensive experience with their conspecifics, regardless of having own offspring or working with children^52^. In this respect, testing children might provide further interesting results.

Furthermore, in contrast to our predictions, participants did not predict aggressive outcomes better than playful or neutral ones. In particular, the outcome of all interactions (i.e. aggressive, playful and neutral) was predicted similarly well for children and NHP. Interestingly, the outcome of aggressive interactions was predicted significantly *worse* than the outcome of playful interactions for dogs. Therefore, although all participants seemed to perform relatively well across conditions for children and NHPs, overall success at predicting dog interactions (Fig. 1) appeared to mainly depend on success at predicting playful interactions (Fig. 2). These results are in line with previous studies using dog pictures/videoclips as stimuli, showing that playful/happy facial expressions are more easily recognized than others (e.g.^41,46^), which might lead children to even misjudge aggressive displays in dogs^47^ (but see^45^), with clear consequences for policies of bite prevention in dogs^53,54,57,58^. Although the co-evolution hypothesis predicts a general increase in human ability to read dog emotions and predict their behaviour (see e.g.^21–23,25^), it is noteworthy that humans, through evolution, mainly selected dogs which were more cooperative and less aggressive^23,77,78^. Perhaps, humans evolved an increased ability to read dog positive, cooperative behaviour, rather than dog behaviour in general. Future studies are needed to gain more insight into the mechanisms of human’s ability to interpret different types of dog behavior.

Finally, our study showed that participants with more experience on a species were more confident about the accuracy of their performance. However, such confidence was unsubstantiated, because it failed to predict their performance with the species. These results have clear implications for policies aiming to reduce the risk of injuries when interacting with dogs, NHP or other taxa, as experience with a species (e.g. dog ownership) might provide an unmotivated confidence, and thus result in riskier human behaviour and an increase in potential injuries (see e.g.^56,73,79,80^).

To our knowledge, this is the first study testing the effect of experience on the ability to predict the outcome of social interactions in three different species, using a controlled and more naturalistic approach (i.e. using videos of real interactions, rather than still pictures; and requiring participants to predict the outcome of interactions, rather than recognize their emotions). Overall, our study provided (i) no support to the co-domestication hypothesis in predicting cross-species social interactions, (ii) evidence of a limited effect of experience on the ability to predict the outcome of social interactions (with a positive effect in NHP, but not in dogs or children), and (iii) no evidence that the outcome of aggressive interactions is better predicted than the outcome of playful or neutral interactions. Based on the finding of the current studies, future studies should consider including more NHP species (and/or participants with different degrees of species-specific experience with NHPs), and also assess the influence of cultural factors (i.e. cultural milieu) on the ability to read their emotions, as it has been done with dogs^49^. Moreover, future studies should incorporate a higher number of participants (especially NHPs, whose sample size was especially limited in our study). Ideally, one should include participants with different types of experience with NHPs, by for instance testing participants living close to NHPs, but not working with them. For instance, the specific type of experience a person had with a species and the exact duration of this experience might both affect the ability to predict the outcome of social interactions. Furthermore, it would be interesting to assess how participants’ performance varies depending on the intensity of aggressive interactions. Possibly, participants would significantly increase their performance when observing interactions with more intense aggression. Finally, to gain a deeper understanding of the processes involved, it would be crucial to determine whether certain body parts of the interacting individuals in the dyad are more salient than others, and how this could influence our ability to read emotions and predict animals’ behaviours (see^45^). In addition, it could be of special interest within the framework of animal injuries prevention to control whether some body parts are more salient and thereby kept longer in the focus of attention than others, depending on the species observed or even the experience and the age of participants^81,82^.

## Supplementary information

Supplementary Video 1.

Supplementary Data.

Supplementary Video 2.

Supplementary Video 3.
